# The use of liquid chromatography-tandem mass spectrometry in newborn screening for congenital adrenal hyperplasia: improvements and future perspectives

**DOI:** 10.3389/fendo.2023.1226284

**Published:** 2023-10-02

**Authors:** Mark de Hora, Natasha Heather, Dianne Webster, Benjamin Albert, Paul Hofman

**Affiliations:** ^1^ Newborn Screening, Specialist Chemical Pathology, LabPlus, Auckland City Hospital, Auckland, New Zealand; ^2^ Liggins Institute, University of Auckland, Auckland, New Zealand; ^3^ Clinical Research Unit, Liggins Institute, University of Auckland, Auckland, New Zealand

**Keywords:** congenital adrenal hyperplasia, newborn screening, steroid profiling, bloodspots, LCMSMS congenital adrenal hyperplasia, LCMSMS

## Abstract

Newborn screening for congenital adrenal hyperplasia using 17-hydroxyprogesterone by immunoassay remains controversial despite screening been available for almost 40 years. Screening is confounded by poor immunoassay specificity, fetal adrenal physiology, stress, and illness which can result in a large number of false positive screening tests. Screening programmes apply higher screening thresholds based on co-variates such as birthweight or gestational age but the false positive rate using immunoassay remains high. Mass spectrometry was first applied to newborn screening for congenital adrenal hyperplasia over 15 years ago. Elevated 17-hydroxprogesterone by immunoassay can be retested with a specific liquid chromatography tandem mass spectrometry assay that may include additional steroid markers. Laboratories register with quality assurance programme providers to ensure accurate steroid measurements. This has led to improvements in screening but there are additional costs and added laboratory workload. The search for novel steroid markers may inform further improvements to screening. Studies have shown that 11-oxygenated androgens are elevated in untreated patients and that the adrenal steroidogenesis backdoor pathway is more active in babies with congenital adrenal hyperplasia. There is continual interest in 21-deoxycortisol, a specific marker of 21-hydroxylase deficiency. The measurement of androgenic steroids and their precursors by liquid chromatography tandem mass spectrometry in bloodspots may inform improvements for screening, diagnosis, and treatment monitoring. In this review, we describe how liquid chromatography tandem mass spectrometry has improved newborn screening for congenital adrenal hyperplasia and explore how future developments may inform further improvements to screening and diagnosis.

## 1 Introduction

Congenital adrenal hyperplasia (CAH) caused by mutations in CYP21A2 results in reduced activity of 21-hydroxylase, an enzyme essential to the synthesis of aldosterone and cortisol. If not detected, the severest form of CAH, leads to life-threatening salt-wasting (SW-CAH) and hypoglycaemia in the early neonatal period. Patients with the simple virilising form of CAH (SV-CAH) have sufficient enzyme activity to maintain electrolyte balance under all but the most extreme conditions (1). The metabolic block in CAH diverts adrenal steroidogenesis towards excessive androgen production, stimulated by a lack of negative feedback on the hypothalamus and pituitary glands by cortisol, causing pre-natal virilisation. Without careful clinical management, increased adrenal androgens (and the aromatization to oestrogens) causes rapid post-natal growth, epiphyseal maturation, premature puberty and subfertility in both sexes. Undetected cases also carry a risk, particularly during childhood, of an acute adrenal crisis during periods of fever or infection. A milder non-classic form of CAH results from a partial enzyme deficiency (NC-CAH) and is considered one of the most common recessive inherited disorders (2), with variable degrees of androgen excess which may lead to rapid growth and premature puberty in childhood and subfertility in adulthood (1).

## 2 Newborn screening for CAH

Newborn screening (NBS) for CAH, available in most developed countries, is successful in preventing salt-wasting adrenal crises with the additional benefits of earlier treatment and reversal of incorrect sex assignment. Measurement of bloodspot 17-hydroxyprogesterone (17OHP), the main accumulating adrenal steroids, by high throughput automated immunoassay in the newborn period is usually used as a screening test. Mutation analysis or characteristic steroid profiles in plasma, with or without an adrenal stimulation test, can confirm the diagnosis (1).

The accuracy of screening has been limited by two main confounding factors. Firstly, dynamic changes in the fetal hypothalamic-pituitary-adrenal (HPA) axis in the early third trimester can lead to the accumulation of large quantities of adrenal steroids and their sulfated conjugates in blood that interfere with immunoassays resulting in falsely elevated measurements of bloodspot 17OHP (3). Secondly, the late expression of some adrenal enzymes in babies born before term, and HPA stimulation of steroidogenesis in stressed or ill neonates, increases the blood concentration of 17OHP (4, 5).

Many NBS laboratories use birthweight (BW) or gestational age (GA) adjusted laboratory thresholds for 17OHP immunoassays as there is a negative correlation between GA and BW with 17OHP measurements (6, 7). Additional improvements may be possible with the combined use of BW and GA (8) or with the collection of additional screening samples (9). Another approach is to use a second-tier immunoassay after the removal of interfering metabolites with a non-polar volatile solvent. While reducing the number of falsely elevated results, the positive predictive value (PPV) of CAH screening remains one of the lowest of disorders included in some NBS programmes (10). Indeed, a review of screening in France led to the recommendation for the discontinuation of screening in premature babies due to the unacceptably low PPV (0.4%) of screening and the close clinical monitoring that is available in hospitalised babies (11), while screening has not yet been recommended in the United Kingdom (12) in part due to the limitations of immunoassay.

Newborn screening using 17OHP immunoassays has high sensitivity in identifying SW-CAH, but some cases of SV-CAH and most NC-CAH will not be detected. In a retrospective analysis of 143 cases of CAH identified by newborn screening over 26 years in Sweden, the sensitivity of screening for SW-CAH, SV-CAH and NC-CAH were 100%, 79.7% and 32.4% respectively when molecular analysis was used to define disease classification (13). Higher 17OHP thresholds for babies born before term will improve the PPV of screening but may reduce the screening sensitivity for SV-CAH, although cases will be missed even with low screening thresholds for 17OHP (14, 15). In general, variations in screening accuracy can also be associated with differences in the timing of sample collection (16), the number of repeat samples collected (9, 16), whether second tier testing is used as part of the screening pathway (17) and how different screening programmes define a positive screening test. The most frequent recommended age for screening sample collection is between 24-72 hours, as later collections increase the likelihood of progressive salt wasting prior to screening notification. However, collection of additional later samples, such as occurs routinely in two-screen states, increases the sensitivity of screening for SV-CAH and NC-CAH (9). In recent years, many screening protocols have incorporated second-tier liquid chromatography-tandem mass spectrometry testing, which accurately measures multiple informative steroids simultaneously and can further improve the efficiency of screening (15, 18, 19).

## 3 Blood spot steroids by liquid chromatography –tandem mass spectrometry

Tandem mass spectrometry (LCMSMS) is a core analytical technology in many clinical and public health laboratories. The technique facilitates the simultaneous quantitation of low molecular weight metabolites in biological specimens. In NBS, target metabolites are extracted from punched bloodspot disks with a suitable volatile polar solvent, then nebulized and ionised through a heated high voltage probe. The resulting molecular ions enter the mass spectrometer in a gaseous state for mass filtering and detection. Mass detection is usually in the multiple reaction monitoring (MRM) mode due to the high signal to noise ratios that can be achieved. Molecular ions are focused through the first mass filter, then fragmented, after which specific mass fragments associated with the molecular ion can be detected and measured. Rapid switching of both mass filters allows specific molecular to fragment ion transitions to be collected for each target metabolite. For steroid analysis, additional sample clean up using liquid chromatography removes ion suppressing compounds that are present in bloodspots, enabling the sensitive measurement of nanomolar concentrations of steroids. The use of matched isotopic labelled steroids improves the accuracy of measurement as they can be used to correct for any loss of target steroids during the sample preparation procedure.

Improvements in NBS for CAH have been possible with the introduction of LCMSMS to measure steroids in bloodspots (18–20). Commonly measured steroid metabolites such as 17OHP, androstenedione (A4), 11-deoxycortisol (11DF), 21-deoxycortisol (21DF) and cortisol (F) are particularly suited to LCMSMS analysis. These and other steroids with a Δ4 ring structure (4-pregnene or 4-androstene) are proton acceptors and are readily ionised using the electrospray technique universally used by newborn screening laboratories. Other informative adrenal steroids in the Δ5 pathway such as dehydroepiandrosterone (DHEA) are more difficult to ionise while testosterone (T) and dihydrotestosterone (DHT) are not adrenal specific and are normally not used in screening protocols.

Early LCMSMS methods described the measurement of 17OHP, A4 and F in bloodspots as a second-tier test on the same bloodspot specimen when the initial 17OHP immunoassay measurements were elevated (19). Steroids are extracted from bloodspots using a solvent such as acetonitrile, methanol or diethylether after which eluants are dried and reconstituted in a suitable solvent for LCMSMS or undergo further purification using an additional solid phase extraction step (21, 22) before analysis. Additional informative steroids such as 11DF and 21DF were incorporated and methods now have established screening protocols using 17OHP, 21DF and a combination of steroid ratios (18, 20). The metabolic block in CAH leads to accumulation of blood 17OHP, A4 and 21DF while the distal metabolites 11DF and F will be reduced. Several methods have incorporated an expanded profile that includes other Δ4 steroids such as progesterone, corticosterone, 11-deoxycorticosterone, testosterone and cortisone but there is no indication that these have been incorporated into NBS protocols (23, 24). Most detailed published methods (**Supplementary Table**) use standard C18 reverse phase chromatography columns which are particularly suited to separate informative steroids in less than 10 minutes (18–21, 23–30).

Accurate measurement is made possible with the incorporation of bloodspot steroid calibrators. Whole blood is washed with saline to remove endogenous steroids and then enriched with known quantities of target steroid metabolites in a serum substitute to manufacture bloodspot calibrators (19, 20, 24). In 2006, a pilot proficiency scheme was made available by the Newborn Quality Assurance Programme provided by the Center for Disease Control and Prevention (CDC, Atlanta, USA). The scheme distributed bloodspots enriched with known quantities of 17OHP, A4 and F to each participating laboratory. Participation in the scheme resulted in improved analyte recoveries and enhanced sample preparation and continuous improvements to second tier testing for CAH (31). The scheme has now expanded to include 11DF and 21DF while participation in the United States has grown from 11 in 2009 to 17 in 2019. Newborn screening for CAH is now available in Australia and the Royal College of Pathologists of Australasia has introduced a pilot scheme for bloodspots steroids to assist six regional laboratories in assessing their performance. Use of short analytical chromatography columns (≤50mm) can speed up analysis times (26) that may be sufficiently rapid for urgent analysis. Several studies have performed prospective and retrospective analyses of bloodspots to determine newborn reference intervals for steroids included in methods (20, 23, 24) but ranges are highly variable due to age, BW and GA ranges that are applied. There remains an ongoing need for the development of local population reference ranges after assay validation.

## 4 Use of LCMSMS to improve screening accuracy

The primary goal of bloodspot steroid analysis in NBS for CAH is to reduce the number of false positive tests encountered using immunoassay while maintaining screening sensitivity. False positive tests result in increased healthcare costs and can lead to lasting anxiety in families (32). LCMSMS is not suitable as a primary screening test as samples are processed sequentially and the cost of dedicated instrumentation is high. The method is almost universally used as a second-tier test when first tier immunoassay 17OHP measurements are elevated.

Analysis of 17OHP by LCMSMS in residual bloodspots revealed that measurements were lower in NBS specimens when compared to immunoassay and that correlation between the methods of measurement was poor (19, 20) due to the improved analytical specificity of LCMSMS. In a retrospective study from Minnesota, measurement of 17OHP by LCMSMS reduced the false positive rate by 55% due to the more specific nature of the second-tier analysis (18). However, prospective data over a 3-year period from the same screening programme, revealed that 41% of specimens reflexed to second tier LCMSMS had 17OHP measurements above the laboratory threshold for a positive screening test (33), indicating a need for alternative approaches to improve the positive predictive value.

One suggested approach is to use the steroid ratio (17OHP+A4)/F to further distinguish between babies with CAH and unaffected newborns. When used in combination, 17OHP and (17OHP+A4)/F, was modelled to reduce the FP rate by 93% in Minnesota (USA) when compared to an immunoassay only approach (18, 19). Similar improvements in the Utah (USA) screening programme found that the false positive rate was reduced from 2.6% to 0.09% (94% improvement) when using 17OHP with (17OH+A4)/F as second tier markers (34). In an Australian prospective study using 2 years of screening data, the positive predictive value of screening was 71.4% with the combined use of 17OHP and (17OHP+A4)/F (28). The use of BW or GA adjusted screening cut-offs for both 17OHP and (17OHP+A4)/F remain necessary as both parameters show a negative correlation with both GA and BW (8, 29). Incorporation of 11DF and 21DF measurements facilitated the calculation of additional informative steroid ratios such as 17OHP/11DF and (17OHP+21DF)/F (20, 21). Most studies showed in improvements in specificity without any loss of screening sensitivity, however retrospective analysis of screening data from Minnesota revealed a reduction in screening sensitivity when second tier LCMSMS was introduced, partly due to the selection of screening thresholds for LCMSMS (14). It should be noted that 11DF and F are not stable when stored at room temperature for extended periods. The use of LCMSMS analysis of stored NBS specimens to set steroid ratio parameter screening threshold such as (17OHP+A4)/F or 17OHP/11DF may lead to inappropriately high thresholds that impact screening accuracy (35).

Incorporating 21DF as an additional bloodspot marker offers further improvements to screening for CAH as 21DF has long been recognised as a sensitive and specific marker of 21-hydroxylase deficiency (36, 37). In CAH, accumulating 17OHP is predominantly converted to 21DF by adrenal specific cytochrome P450 11β-hydroxylase (CYP11B1). In a retrospective and prospective study from Germany, 21DF showed a clear distinction between CAH affected and unaffected newborns. Additionally, the use of (17OHP+21DF)/F led to further improvements in sensitivity (20). A further benefit of using 21DF is that levels do not appear to correlate with BW or GA (37).

Two screening programmes have evaluated the use of 21DF alone as a second-tier screening marker. In a study from Wisconsin, 906 newborn screening specimens were subjected to 21DF analysis (851 unaffected, 55 affected with CAH) that yielded a test PPV of 91.7% when the laboratory threshold for 21DF was optimised for 100% sensitivity (37). A similar outcome was found in a screening pilot from the Netherlands which found the 21DF eliminated false positive results if used following 17OHP immunoassay (38). In both studies there were mild elevations in 21DF in some specimens from babies that were presumed not to have CAH which would result in a few additional sample recollections.

To date, prospective studies from newborn screening programmes, summarised in **Table 1**, have shown that the use of LCMSMS as a second tier test incorporating 17OHP, steroid ratios and 21DF can improve the PPV of NBS for CAH. Some milder cases of CAH may be missed by this two-tier approach.

**Table 1:** Prospective studies on newborn screening for CAH using second tier LCMSMS analysis.

Author	No Screens	No. 2^nd^ Tier Tests	CAH cases	2^nd^ tier parameters	PPV before LCMSMS	PPV After LCMSMS
Janzen et al 2007 (20)	242,500	1609	16	17OHP (17OHP+21DF)/F	1.0%	100%
Matern et al., 2008 (33)	204,281	1298	9	17OHP (17OHP+A4)/F	0.8%	7.3%
Schwarz et al., 2009 (34)	64,115	1709	6	17OHP (17OHP+A4)/F	<1.0%	9.4%
Dhillon et al., 2011 (25)	2,702,000	10,932	143	17OHP (17OHP+A4)/F	1.3%	7.0%
Seo et al 2014 (39)	5852	104	2	17OHP (17OHP+A4)/F	1.9%	100%
Bialk et al 2019 (29)	63,725	472	5	17OHP (17OHP+A4)/F	1%	17%
Lai et al 2020 (28)	202,960	4218	12	17OHP (17OHP+A4)/F	–	71.4%
Stroek et al 2021 (38)	–	350	37	21DF	24.7%	53%
Cavarzere et al., 2022 (40)	99518	–	3	17OHP, 21DF,11DF (17OHP+A4)/F	0.24%	2.54%
de Hora et al., 2022 (15)	236,835	1915	11	17OHP, 21DF (17OHP+A4)/F	1.7%	45.8%
Lind Holst et al., 2022 (41)	593,435	15121	29	17OHP (17OHP+A4)/F	–	55.8%

## 5 Future directions – bloodspot androgen markers in CAH

### 5.1 Pathways to androgen synthesis in CAH

The enzyme deficiency in CAH diverts adrenal steroidogenesis towards excessive adrenal androgen production. There has been significant progress in our understanding of the pathways to androgen synthesis in CAH that raises the possibility of using less recognised androgen metabolites as biomarkers of CAH (42). In the classic adrenal steroidogenesis pathway, A4 and T are synthesised via DHEA as 17OHP is poorly converted directly to A4 by 17α-hydroxylase/17,20 lyase (CYP17A1). In CAH, accumulating 17OHP may overcome the low 17,20 lyase activity of CYP17A1 and drive the direct conversion of 17OHP to A4. In target tissues T is converted by 5α reductase (types 1 or 2) to the potent androgen dihydrotestosterone (DHT). Additionally, A4 may be 5α-reduced to 5α-androstandione (5αdione) before 17β-reduction by to DHT (43).

**Figure 1:**
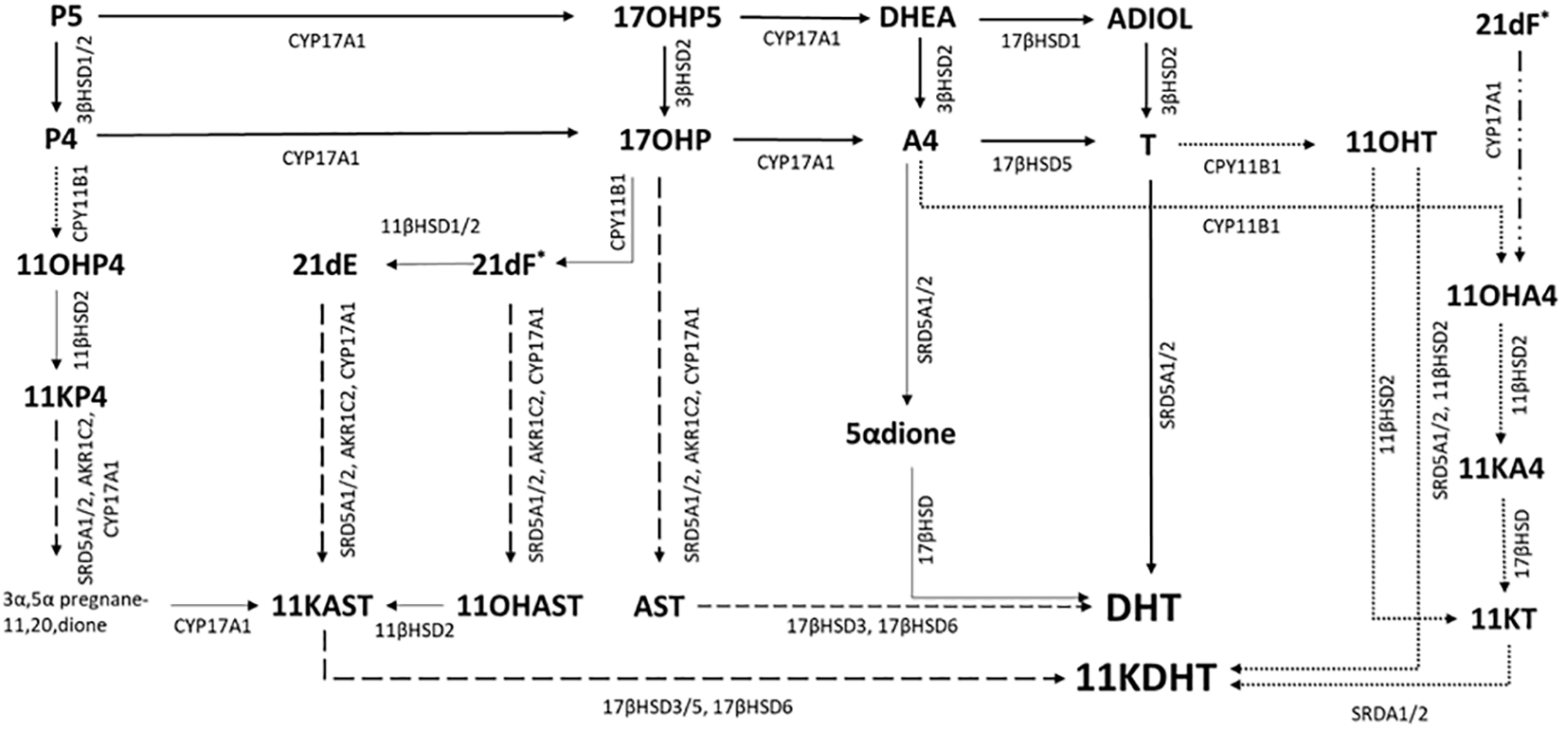
Possible pathways to androgen synthesis in CAH. Steroidogenesis is diverted by the metabolic block in CAH in classic pathway via A4 and DHEA. Accumulating 17OHP can overcome the relatively low A4 substrate affinity for 17α-hydroxylase/17,20 lyase CYP17A1 that results in A4 been directly converted to T. Subsequent 5α reduction (SRD5A) of T to DHT, one of the 2 most potent androgens, occurs in target tissues (Solid arrows). Accumulating 17OHP can also be metabolised to DHT via the steroidogenesis backdoor pathway, after 5α and 3α reduction the 17,20-lyase activity of CYP17A1 forms the C19 steroid androsterone (dashed arrows) which is then converted to DHT by subsequent reductive (17βHSD6) and oxidative reactions (17βHSD3). *In vitro* studies have suggested that additional accumulating C21 steroids, such as 21-deoxycortisol (21dF), 21-deoxycortisone (21dE) and 11-hydroxyprogesterone (11OHP4) can be metabolised by the backdoor pathway to 11-ketodehydrotestosterone (11KDHT), a C19 steroid with similar androgenic potency as DHT (dashed arrows). An additional route for androgen synthesis is through the C19 oxygenated steroid pathway (dotted arrows). Accumulating A4 and T are converted to 11OHA4 and 11OHT via the adrenal specific enzyme CPY11B1. 21DF may also be converted directly to 11OHA4 BY CYP17A1. 11OHA4 is the most abundant 11-oxygenated steroid. KT is synthesised via 11KA4 (11βHSD2, 17βHSD) and subsequently 5^α^-reduced to 11KDHT. Enzymes are denoted by their coding gene. 17βHSD, 17β-hydroxysteroid dehydrogenase; CYP11B1, 11β-hydroxylase; SRDA1/2, 5α-reductase; AKR1C2, aldo-keto reductase.

In 2004, a “backdoor” pathway was described with a metabolic route from 17OHP to DHT that does not involve A4 or T. In CAH, accumulating 17OHP is 5α- and 3α- reduced before being converted to androsterone by CYP17A1 with subsequent reduction and oxidation steps yielding DHT (44). Urinary steroid profiles in babies with CAH revealed that this pathway is active in CAH in the newborn period (45). The backdoor pathway may also make further contributions to the total androgen pool in CAH in the newborn period. *In vitro* studies have demonstrated that 11-hydroxylated corticosteroids such as 21DF, 21-deoxycortisone (21DE) and 11β-hydroxyprogesterone (11βOHP) can be converted by backdoor pathway enzymes to yield 11-ketodihydrotestosterone (11KDHT) (46), an androgen with a similar potency to DHT (**Figure 1**). Almost 60 years ago, Jailer and colleagues demonstrated that 21DF and not 17OHP dosing resulted in increased 11-hydroxyandrosterone (11OHAST) excretion, an indication that 21DF is an androgen precursor (47). Whether the route is via the backdoor pathway or by the direct conversion of 21DF to 11OHA4 via CYP17A1, 21DF may be an important contributor to the androgen pool in CAH.

More recently, there has been growing interest in the role of 11-oxygenated androgens in CAH. The adrenal glands produce a series of C19 steroids via the adrenal specific enzyme, CYP11B1. The most abundant of these 11-oxygenated androgens is 11-hydroxyandrostenedione (11OHA4), which normally circulates at higher concentrations than A4 (48). In CAH, accumulating A4 is readily hydroxylated by CYP11B1 leading to higher circulating concentrations of 11OHA4 and then oxidised in the periphery to 11-ketoandrostenedione (11KA4). Additionally, accumulating T is converted by adrenal CYP11B1 to 11OH-testosterone and then converted to 11-ketotestosterone (KT). Both 11KT and T have similar androgenic potency. Accumulating 11KA4 can be converted to 11KT by 17β-reduction in peripheral tissues (**Figure 1**). At specific target tissues such as adipose, prostate and skin, T, 11OHT and 11KT can be converted to the most potent androgens, DHT and KDHT. In treated classical CAH patients, plasma studies have shown that 11-oxygenated C19 steroids are the dominant circulating adrenal specific androgen precursors (48). Additionally, Turcu and colleagues revealed that adrenal 11-oxygenated androgen are disproportionally elevated compared to T and A4 in non-classical CAH in unstimulated blood tests (49).

### 5.2 Bloodspot androgen measurements for NBS for CAH

The challenge for NBS laboratories is the development of methods that can measure androgens and androgen precursor steroids in bloodspot specimens in a reliable way and to characterize the typical profiles in bloodspots from babies with classical CAH and unaffected newborns. While studies have revealed the 21DF is the most specific single corticosteroid marker for CAH, it is not 100% sensitive or specific in newborn screening (37, 38). The development of methods to include the 11-oxygenated C19 steroids along with DHT and KDHT in newborn screening for CAH may offer further improvements to screening accuracy. The C19 oxygenated steroids will undergo sufficient ionization in LCMSMS due to their 4-androstene structure while the saturated androgens (11KDHT, DHT) and the 5-androstenes (DHEA) have much lower ionization efficiencies that require more sensitive and expensive instrumentation for reliable quantitation.

Alternatively, chemical derivatisation can be used to improve the ionisation of steroids by electrospray ionisation. Many screening laboratories butylate endogenous amino acids and acylcarnitines to increase the ionisation efficiency of target compounds to screen for amino and fatty acid breakdown disorders. Use of derivatisation to enhance the sensitivity of steroid measurements has been reviewed (50, 51) but the most common method for enhancing the sensitivity of Δ5 steroids and C19 androgens is an oximation reaction. This is usually done as a last step in sample preparation and does not require any additional sample clean up after the derivatisation reaction is complete. In a method described by Caron and colleagues, hydroxylamine derivatised C19-oxygenated steroids were measured in plasma at a lower limit of quantification than other non-derivatised methods for 11A4OH, 11KA4, 11OHT, 11KT, 11KDHT, 11OHAST and 11KAST (52). The derivatisation method has also been applied to measure a broader range of steroids including DHT, corticosteroids and the steroids of the Δ5 pathway (53). while methoxylamine derivatisation was used to measure 17 ketosteroids in plasma (54). In a further study, oximation of ketosteroids before LCMSMS improved the lower limit of quantification for DHT by 25-fold when compared to an underivatized approach and greater improvements were achieved for DHEA and 21DF (55).

One of the limitations of derivatising steroids before LCMSMS analysis is that there is no universal derivatising chemical available for all steroid classes. Oximation only targets steroids with a carbonyl group, however almost all informative steroids in CAH have carbonyl functional groups. A second limiting factor of using hydroxylamine as a derivatising reagent is that several isoforms of target steroids can occur as hydroxylamine groups can form in an α or β configuration and steroids with 2 or more carbonyl group generally result in 2 chromatographic peaks. Lastly, isobaric androgen derivatives may also co-elute and chromatographic conditions should be sought to ensure they are appropriately separated on the chosen chromatography column. In a technical report, Hakkinen and colleagues assessed 3 types of reversed phase columns and found that a biphenyl column had enough selectivity to separate most of the ketosteroids that may be informative for NBS for CAH (51).

In summary, the accuracy of newborn screening for CAH due to 21-hydroxylase deficiency is improved with the use of second tier LCMSMS analysis. Screening programmes that use this approach have a lower false positive rate than programmes that use immunoassay alone. The use of additional steroid parameters such as (17OHP+A4)/F and 21DF offer even better sensitivity and specificity while incorporating oxygenated C19 steroids and the potent androgens DHT and KDHT may, in the future, offer further improvements to screening performance. To date, the markedly improved PPV due to the use of LCMSMS has strengthened the case for CAH newborn screening, that may be further strengthened as new markers are incorporated in NBS protocols.

## Author contributions

Conceptualization; MD, DW, NH, BA, PH. Methodology; MD. Formal analysis; MD. Investigation; MD; Resources, MD; Data curation, Original draft preparation; MD. Writing, review and editing; NH, DW, BA, PH. Visualization; MD. Supervision; PH, NH, DW, BA. All authors contributed to the article and approved the submitted version.
