# Supplementary material for: The use of liquid chromatography-tandem mass spectrometry in newborn screening for congenital adrenal hyperplasia: improvements and future perspectives
Source: Front Endocrinol (Lausanne). 2023 Oct 2;14:1226284. doi: 10.3389/fendo.2023.1226284 (PMC10578435; doi:10.3389/fendo.2023.1226284)
Supplement: Supplementary file 1 [file Table_1.docx]

**Supplementary Table** – Blood Steroid Profiling LCMSMS Methods used for Newborn Screening for CAH

| **Method** | **Instrument** | **Chromatography Column** | **Spot Size** | **Extraction Solvent** | **Sample**  **Runtime** | **Internal Standards** | **Target Steroids** |
| --- | --- | --- | --- | --- | --- | --- | --- |
| Lacey J. et al 2004 (19) | SCIEX API 3000 | C18, 2.1 x 20mm | 4.8mm | DEE | 12.0 min | d_8_-17OHP | 17OHP, A4, F |
| Janzen N. et al 2007 (20) | Waters Ultima | C18 3μm, 2.1 x 20mm | 6.0mm | ACN/H_2_O (50:50) | 6.0 min | d_8_-17OHP, d_2_-F | 17OHP, A4, F, 21DF, 11DF |
| Dhillon et al 2011 (25) | SCIEX API 3000 | C18, 5μm, 2.1x50mm | 4.7mm | MeOH/ H_2_O (90:10) | 6.5 min | d_8_-17OHP, d_4_-F,  d_7_-A4 | 17OHP, A4, F |
| Janzen et al 2011  (26) | Waters Xevo | C18, 1.7μm, 2.1x50mm | 2x4.7mm | ACN/ACE (50:50) | 1.5 min | d_8_-17OHP, d_8_-21DF d_2_-F d_2_-11DF d_7_-A4 | 17OHP, A4, F, 21DF, 11DF |
| Magnisali et al 2011 (21) | SCIEX API 5000 | C18, 5μm, 2.1x50mm | 5mm | MeOH (followed by SPE) | 3.0 min | d_8_-17OHP, d_4_-F | 17OHP, A4, F, 11DF, E |
| Rossi et al 2011  (27) | Waters Xevo | C18, 1.8μm, 2.1x50mm | 2x3.0mm | ACN/ACE (50:50) | 3.5 min | d_8_-17OHP, d_4_-F  d_7_-A4 | 17OHP, A4, F |
| Kim et al 2015 (23) | Agilent 6490 | C18, 2.6μm, 2.1x50mm | 3.2mm | MeOH/ACN (50:50) | 20 min | d_8_-17OHP, d_4_-F  d_2_-11DF d_8_-21DF d_7_-A4 d_8_-B d_8_-11DOC | 17OHP, A4, 21DF, 11DF, F, B, 11DOC |
| Boelen 2016 (24) | Waters Xevo TQS | C18, 1.7μm, 2.1x50mm | 2x3.2mm | ACN/ H_2_O (80:20) | 6.5 min | d_8_-17OHP | 17OHP, A4, T, 21DF, 11DF, 11DOC, E, F, B |
| Lai 2020 (28) | Waters Xevo TQS | C18, 1.8μm, 2.1x50mm | 3.2mm | MeOH/ H_2_O (95:5) | 3.5 min | d_8_-17OHP, d4-F d_7_-A4 | 17OHP, F, A4 |
| Bialk 2019 (29) | SCIEX API 5000 | C18, 5μm, 2.1x50mm | 3.2mm | ACN/H_2_O (80:20) | 9.0 mins | d_8_-17OHP, d_8_-21DF d_4_-F d_7_-A4 | 17OHP, A4, 21DF, 11DF, F |
| Zhan 2022 (30) | Waters Xevo TQS | C18, 1.7μm, 2.1x50mm | 2x5mm | ACN/MeOH (50:50) | 8.0 mins | d_8_-17OHP, d_4_-F d_2_-11DF d_3_-T d_3_-A4 | 17OHP, A4, 21DF, 11DF, F, T |
